# Therapeutic potential and mechanisms of sacral nerve stimulation for gastrointestinal diseases

**DOI:** 10.2478/jtim-2023-0086

**Published:** 2023-07-05

**Authors:** Ximeng Wang, Jiande DZ Chen

**Affiliations:** Department of Biomedical Engineering, Johns Hopkins University, Baltimore, MD 21228, USA; Department of Internal Medicine, University of Michigan School of Medicine, Ann Arbor MI 48109, USA

**Keywords:** sacral neuromodulation, sacral nerve stimulation, autonomic functions, inflammatory bowel disease, functional gastrointestinal diseases

## Abstract

**Background:**

The aim of this systemtic review is to introduce clinical applications (especially emerging) and potential mechanisms of sacral nerve stimulation (SNS) for treating various gastrointestinal diseases.

**Materials and Methods:**

PubMed and Web of Science were searched for studies published on SNS and its clinical applications in fecal incontinence (limited to systematic review and meta-analysis of clinical studies), constipation (limited to reviews and randomized control clinical studies), irritable bowel syndrome (IBS), inflammatory bowel disease (IBD) and upper gastrointestinal motility disorders. The relevant studies were pooled, and their findings were summarized and discussed.

**Results:**

SNS is an approved method for treating fecal incontinence. Systematic review and meta-analysis demonstrated high efficacy of the SNS therapy for fecal incontinence. Increased anal sphincter pressure and improvement in rectal sensation were reported as major mechanisms involved in the SNS therapy. SNS has also been proposed for treating constipation, but the therapy has been shown ineffective. There is a lack in SNS methodological optimization and mechanistic research. A few basic and clinical studies have reported the potential of SNS for treating visceral pain in IBS. SNS seemed capable of improving mucosal barrier functions. Several case reports are available in the literature on the treatment of IBD with SNS. Several laboratory studies suggested therapeutic potential of a special method of SNS for IBD. Cholinergic anti-inflammatory mechanisms were reported. Due to a recently reported spinal afferent and vagal efferent pathway of SNS, a few preclinical studies reported the potential of SNS for upper gastrointestinal motility disorders. However, no clinical studies have been performed.

**Conclusions:**

SNS for fecal incontinence is a well-established clinical therapy. However, the current method of SNS is ineffective for treating constipation. Further methodological development and randomized clinical trials are needed to explore potential applications of SNS for IBS and IBD.

## Introduction

**Figure 1:**
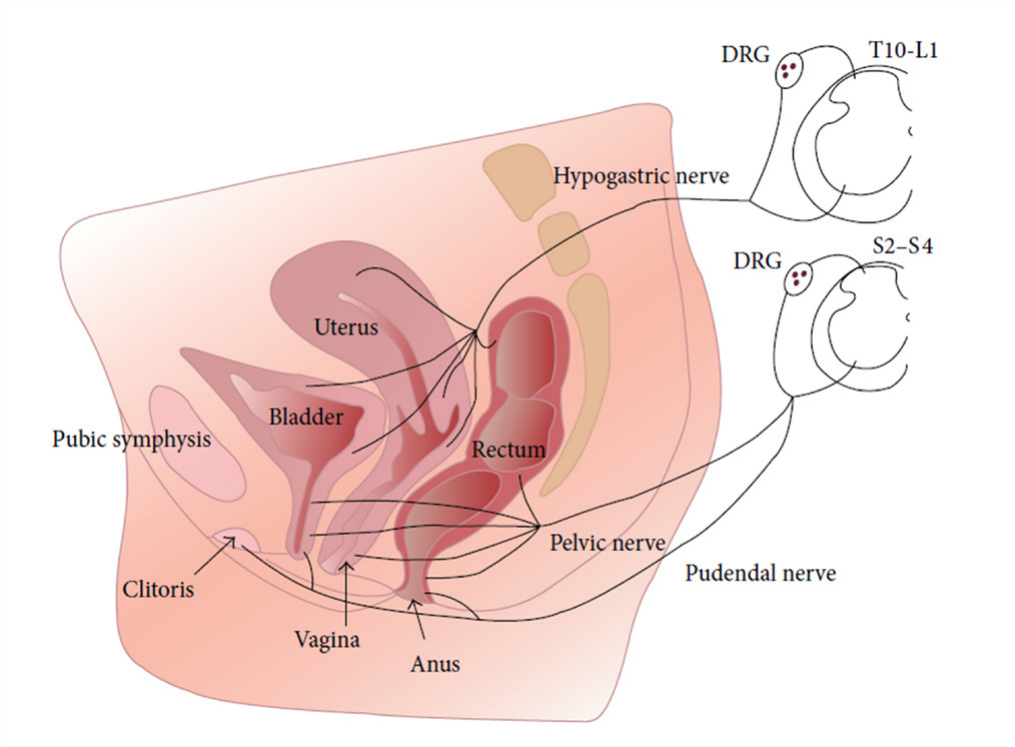
Innervation of the pelvic organs. The parasympathetic nervous system includes vagus nerve and sacral nerve. The vagus nerve innervates peripheral organs in the proximal part of the body, such as heart, lung, liver, stomach and small intestine, whereas the sacral nerve innervates pelvic organs such as rectum, anus, vagina and bladder *via* the pelvic nerve. This direct innervation provides opportunities for the use of sacral nerve stimulation for treating disorders of these organs. Reproduced from Jobling *et al*., 2014^[1]^. DRG: dorsal root ganglion.

The parasympathetic nervous system includes the vagus nerve and the sacral nerve. The vagus nerve innervates peripheral organs in the proximal part of the body, such as heart, lung, liver, stomach and small intestine. The sacral nerves innervate pelvic organs, including colon, anorectum, bladder and genital organs in addition to the lower extremities^[1]^ (Figure 1). These nerves lie on the back of the pelvis in front of the piriformis muscle and the pelvic fascia. In rats, the majority of the sacral nerve fibers were reported to be unmyelinated with a ratio of unmyelinated to myelinated fibers of 1.66 for the S3-S4 roots.^[2]^ The majority of fibers in the sacral nerve are afferent; it was reported that the human S3, S4 and S5 dorsal roots contained 3, 18, and 20 to 30% efferent fibers respectively.^[3]^

Sacral nerve stimulation (SNS) has been introduced or explored for the treatment of disorders of these pelvic organs; it is also called sacral neuromodulation in some publications. SNS received the Food and Drug Administration (FDA) approval in

1997 for treating overactive bladder that affects more than 15% of people in the US.^[4,5]^ A recent meta-analysis revealed a success rate of 58.5% with the SNS treatment in patients with overactive bladder who failed the botulinum toxin A therapy.^[6]^

Inspired by the success in treating urinary disorders, SNS has also been applied for treating functional gastrointestinal diseases, such as fecal incontinence, constipation and irritable bowel syndrome (IBS). Using a similar method as for urinary disorders, SNS was applied for treating fecal incontinence.^[7]^ and later received the FDA approval. However, the clinical outcomes with the application of the same method of SNS for constipation have been controversial.^[8,9]^ SNS for constipation received Conformite Europeenne (CE) Mark in Europe (fulfilling the requirements of relevant European product directives and harmonized performance and safety standards) but failed to receive the FDA approval in the US. Recently, investigators also explored the clinical application of SNS for IBS with initial success.^[10]^

In addition, the potential of SNS for treating other gastrointestinal diseases have also been investigated in preclinical studies. A number of preclinical studies have shown promising results in the use of SNS with specific set of stimulation parameters and treatment regimens for treating intestinal inflammation.^[11,12]^ The most surprising and interesting finding was that the anti-inflammatory effect of SNS was largely mediated *via* the spinal afferent and vagal efferent pathway as bilateral vagotomy substantially abolished the SNS-induced effects.^[13]^ This novel pathway was also supported by the fact that the anti-inflammatory effect of SNS was nearly identical to the cervical vagal nerve stimulation (VNS) and the combination of SNS and VNS.^[14]^

To further confirm the existence of such spinal afferent and vagal efferent pathway, experiments were designed to investigate whether SNS was able to alter or improve motility functions of the stomach that is not innervated with the sacral nerve and the study outcomes demonstrated that SNS was indeed able to change/ improve gastric motility functions.^[15,16]^ These early findings suggest that SNS may also be used to treat certain disorders of an organ that is innervated with the vagal nerve but not the sacral nerve.

The aim of this review is to provide an overview on the applications of SNS for treating functional gastrointestinal diseases and inflammatory bowel diseases including methodologies, therapeutic potential, mechanisms and perspectives.

## Methods and Materials

This is an expert opinion-based mini review. The review is specifically focused on emerging potential of SNS in the area of gastroenterology. The literature search was performed on PubMed and Web of Science (all available years) using the following phrases “sacral nerve stimulation” or “sacral neuromodulation” plus other specific phrases associated with the subject of each section, such as “fecal incontinence”, “constipation” or “irritable bowel syndrome”. Since most SNS applications in gastroenterology were new, clinical studies in pediatric patients were excluded.

Varying inclusion and exclusion criteria were used for different sections of the review, as described below.

Section on SNS for Fecal Incontinence. Since the therapy of SNS for fecal incontinence is well established clinically, only following clinical studies were included in the review: clinical study reports, recent systematic reviews with large sample size (100 patients or more). Studies on the treatment of specific disease categories of fecal incontinence were excluded.

Section on SNS for Constipation: recent systematic reviewers were assessed by the authors and included in the review. Although there was a large number of clinical studies in the literature, only a few studies were randomized and controlled (included in the review). Individual uncontrolled clinical studies were excluded.

Section on SNS for IBS: There was only a small number of clinical studies and all of them were assessed and reported in the review. Since this review was focused on emerging applications of SNS, relatively more information was provided on individual clinical studies.

Section on SNS for inflammatory bowel disease (IBD): This is a new area of research with limited clinical studies. To introduce the potential of SNS for IBD, the PubMed and Web of Science search included all available animal and clinical studies.

Section on SNS for Upper gastrointestinal (GI) Disorders: This is an emerging area of research. The search included all available animal and clinical studies.

## SNS for Functional GI Diseases

### SNS for fecal incontinence

Fecal incontinence is a common symptom and was reported to affect about 7% to 15% of people in community-dwelling men and women.^[17]^ It is more common in older adults and its prevalence is between 50% to 70% in those who are in nursing homes.^[17]^ The main pathophysiological factors include reduced anal sphincter pressure and altered rectal sensation and compliance.^[18]^

#### Method of sacral nerve stimulation

**Figure 2:**
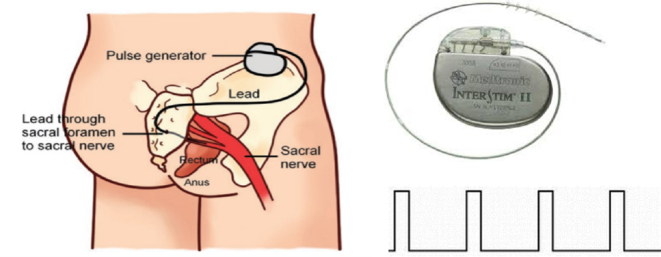
Sacral nerve stimulation for fecal incontinence. Left: embodiment of the stimulation lead and pulse generator; top right: Implantable pulse generator connected with stimulation lead from Medtronic Inc; bottom right: continuous stimulation pulses used for treating fecal incontinence.

SNS is accomplished by delivering electrical stimulation to the sacral nerve *via* a pair of electrodes chronically implanted at the sacral nerve (S3) *via* an implantable pulse generator (IPG) surgically placed in the buttock (Figure 2). Under general anesthesia, the SNS lead is inserted into the S3 sacral foramen under X-ray guidance. It typically contains 4 electrodes on its tip and two of the electrodes are selected for applying bipolar stimulation based on their proximity to the sacral nerve. The IPG is surgically implanted underneath the skin in a convenient location in the buttock. It is powered by either a primary battery or a rechargeable battery. The setting of the IPG is controlled by an external controller that can send a set of programmed stimulation parameters to the IPG. While a caring physician has full control of the device, such as various parameter settings and electrode configuration, a patient is able to control only the stimulation intensity within a safe limit.

The first clinical study of SNS for treating patients with fecal incontinence used the following stimulation parameters: a pulse frequency of 15 Hz, pulse width of 210 μs, intermittent stimulation with an on-off ratio of 5:1 sec and output at a voltage of 1 to 10 V based on the patient’s perception and on muscular contractions of the perineum and anal sphincter.^[7]^ The stimulation was performed 24 h per day.

The temporary SNS stimulation, commonly termed as the peripheral nerve evaluation (PNE), is an optional procedure before implanting the IPG for the long-term SNS treatment. During the PNE, SNS is performed *via* an external stimulation device. The suggested duration for PNE is 2-4 weeks based on a recent European consensus statement.^[19]^ At the end of the PNE, the patient undergoes a second procedure under general anesthesia for the placement of a permanent IPG, if the outcome of the PNE is satisfactory (50% improvement in symptoms) or the removal of the stimulation electrode lead, if the outcome is not satisfactory.

#### Clinical efficacy of SNS for fecal incontinence

Clinically, SNS is indicated for the treatment of chronic fecal incontinence in patients who have failed or are not candidates for other treatments, such as medications or physical therapy.^[20]^ The success of SNS for fecal incontinence is defined as a reduction of more than 50% in fecal incontinence episodes. The literature is rich in clinical studies investigating the efficacy of SNS for fecal incontinence. There were 21 systematic reviews and/ or meta-analysis on the efficacy of SNS for fecal incontinence on PubMed and Web of Science since 2004. Large clinical studies (100 patients or more) reported the effectiveness of chronic SNS;^[21,22]^ the success rate was 80% with medium to long term SNS therapies based on a recent systematic review.^[23]^ Table 1 summarized the effects of SNS in various clinical studies published in 2013 or earlier.^[23]^ Further improvement in SNS methodologies, such as stimulation parameters and treatment regimens and/or more appropriate selection of patients are expected to yield better treatment outcomes.^[24]^ From the published data so far, there does not seem to have an issue of tachyphylaxis, which is encouraging.

**Table 1:** Effects of sacral nerve stimulation for treating fecal incontinence.

	Permanent Implants				Intention-to-treat			
References	Median follow-up (months)	No. at baseline	No. at follow-up	%. at follow-up	> 50% improvement in FI episodes per week (%)	100% continence (%)	> 50% improvement in FI episodes per week (%)	100% continence (%)
Short-term follow-up (≤ 12 months)								
Jarrett *et al*.^[46]^	12	12	12	100	n.a.	42	n.a.	8
Leroi *et al*.^[78]^	6^Ɨ^	34	19	56	n.a.	26	n.a.	n.c.
Gourcero *et al*.^[49]^	12	33	29	88	69	21	33	10
Tjandra *et al*.^[38]^	12^Ɨ^	53	53	100	71	47	63	42
Boyle *et al*.^[56]^	6	13	13	100	77	46	66	40
Dudding *et al*.^[82]^	8	30	30	100	80	40	n.c.	n.c.
Hollingshead *et al*.^[61]^	12	86	86	100	81	n.a.	62	n.a.
Wexner *et al*.^[65]^	12^Ɨ^	120	106	88	83	41	66^ǂ^	32
Santoro *et al*.^[76]^	6^Ɨ^	28	28	100	n.a.	68	n.a.	68
Medium-term follow-up (12-36 months)								
Kenefick *et al*.^[40]^	24	15	15	100	n.a.	73	n.a.	73
Matzel *et al*.^[3]^	24	34	34	100	88	35	81	32
Kenefick *et al*.^[48]^	24	19	19	100	n.a.	74	n.a.	74
Melenhorst *et al*.^[52]^	26^§^	100	100	100	79	n.a.	59	n.a.
Dudding *et al*.^[11]^	24	51	48	94	65	40	52	32
Munoz-Duyos *et al*.^[15]^	35	29	29	100	85	48	58	33
Govaert *et al*.^[57]^	31^§^	145	145	100	80	n.a.	56	n.a.
Govaert *et al*.^[58]^	35^§^	173	169	98	77	n.a.	53	n.a.
Kock *et al*.^[62]^	24^§^	19	19	100	n.a.	21	n.a.	11
Oom *et al*.^[63]^	32	37	37	100	81	5	65	4
Mellgren *et al*.^[70]^	36^Ɨ^	120	77	64	86	40	59^ǂ^	26
Boyle *et al*.^[72]^	17	37	37	100	73	35	54^ǂ^	26^ǂ^
Long-term follow-up (> 36 months)								
Altomare *et al*.^[55]^	74^§^	60	52	87	n.a.	18	n.a.	10
Dudding *et al*.^[82]^	51	18	18	100	94	39	n.c.	n.c.
Matzel *et al*.^[17]^	118	12	9	75	78	44	58	33
Vallet *et al*.^[59]^	44^§^	32	23	72	n.a.	4	n.a.	2
Hollingshead *et al*.^[61]^	60	86	18	21	83	n.a.	n.c.	n.c.
Lombardi *et al*.^[84]^	46^Ɨ^	11	11	100	100	27	n.c.	n.c.
Duelund-Jakobsen *et al*.^[71^	^]^ 46	158	91	58	75	36	n.c.	n.c.
Uludag *et al*.^[90]^	85	50	50	100	84	n.a.	n.c.	n.c.
Devroede *et al*.^[74]^	48	120	77	64	87	34	50	20
George *et al*.^[75]^	114	23	19	83	n.a.	52	n.a.	48
Summary*								
Short term	12(6-12)		29106(12) -	100100() 56-	79(69-83)	42(21-66)	63(33-66)	36(6-66)
Medium term	25(17-36)		37169(15) -	100100() 64-	80(65-88)	40(5-74)	58(52-81)	32(4-74)
Long term	56118(44) -		21(9-91)	79100(21) -	84(75-100)	35(4-52)	54(50-58)	20(2-48)

All values have been calculated to the nearest integer. * Values are median(range); ^Ɨ^ value at specific point; ^ǂ^ intention-to-treat reported by study; ^§^ mean value. FI: faecal incontinence; n.a.: data not available; n.c.: not calculable. References and the numbers of references can be found in the systematic review paper (Thin et al., 2013^[23]^).

The safety profile of SNS for fecal incontinence is good. Reported adverse events included pain in the implantation site, particularly in slimmer patients, seroma, excessive tingling in the pelvic region, infection; None of these incidences is in excess of 15%.^[25,26]^

#### Mechanisms of SNS for fecal incontinence

Since the method of SNS for fecal incontinence was adopted from the SNS method used for urinary disorders, not much is known about the mechanisms involved in the SNS therapeutic effect on fecal incontinence. However, available clinical data have demonstrated an increase in anal sphincter pressure and improvement in rectal sensation with chronic SNS, the two major physiological factors controlling the continence.^[27, 28, 29, 30]^

As we stated earlier, the anal sphincter is critical in maintaining the continence of the rectum. The SNS-induced increase in anal sphincter pressure is suggestive of enhanced striated anal sphincter muscle activities.^[31]^ It was postulated that the long-term SNS might be able to transform fast twitch fatigable muscle into slow twitch fatigue-resistant fibers,^[32,33]^ enabling the patient to maintain a sustainable duration of squeeze to avoid incontinence. The anal sphincter is composed of an internal sphincter and an external sphincter. While the internal sphincter is involuntarily controlled and relaxed upon rectal filling of feces, the external sphincter can be consciously controlled. The voluntary control is based on the sensation of the rectal filling or distention. Impairment in rectal sensation, such as rectal hyposensitivity, results in fecal incontinence. A few clinical studies reported improvement in rectal sensation to rectal distention with SNS,^[28,34]^ possibly attributed to enhanced afferent sensory functions as observed in animal studies.^[35,36]^

### SNS for constipation

Chronic constipation is a common disorder, affecting about 16% of adults worldwide; it is more common in female and adults over 60 years of age.^[37, 38, 39]^ Major symptoms of constipation include a reduced frequency of bowel movement (< 3 times/week), hard stools and strain during defecation. Constipation can be classified into slow transit constipation, defecatory disorder and normal transit constipation; lack of rectal sensation is also believed play an important role.^[40]^ Common treatment methods include dietary fiber supplements, laxatives and prokinetic agents as well as biofeedback training (for defecatory disorder).^[41]^ Surgical resection of the colon or a segment of the colon is the last option for patients with severe constipation refractory to medical therapies.^[41]^

SNS was introduced to treat patients with constipation more than 30 years ago.^[42]^ The surgical procedure, device (electrode lead and IPG) and stimulation parameters used for constipation are the same as those for fecal incontinence. The PubMed and Web of Science search revealed about 100 clinical studies on SNS for constipation and 8 reviews. Recent systematic review and meta-analyses indicated ineffectiveness of SNS for constipation.^[9,43, 44, 45]^ Overall, the evidence to support the clinical efficacy of SNS for constipation was low due to small sample size and uncontrolled nature of the clinical study design. ^[46]^ A recent review indicated that stimulation was associated with clinical and statistically meaningful improvement in symptoms of constipation.^[47]^ In a placebo controlled clinical study with relevant outcome measures in 53 patients, a 3-week SNS treatment was reported to be ineffective in treating constipation.^[48]^ A long-term follow up study confirmed the same results.^[49]^ These findings indicated that the particular method of SNS, *i.e*., a stimulation frequency of 14 Hz and pulse width of 300 μs, was ineffective in treating patients with slow transit constipation.

Although the potential of SNS for treating constipation has been explored in more than 100 clinical studies, there was a lack of optimization of the SNS therapy for constipation. Unfortunate and surprisingly, the SNS method used for constipation in a vast majority of the clinical studies was adopted from the method used for fecal incontinence and overactive bladder, including the stimulation parameters and treatment regimens (continuous 24 h per day stimulation). Although the SNS method applied successfully for treating fecal incontinence was also copied from the SNS method for overactive bladder, it was because there are similarities in their pathophysiologies between the overactive bladder and fecal incontinence, such as hyperactivity and impaired sensation. Constipation is, however, largely attributed to hypoactive colon or hypomotility, which is different from fecal incontinence or overactive bladder. Therefore, conceptually, a different method is needed to treat constipation, such as activation of colon motility.

In a recent rodent study performed in our lab, we used a set of SNS parameters derived based on its effect on rectal tone that was totally different from that used for treating constipation/ fecal incontinence; SNS with such a set of parameters enhanced vagal activity, released acetylcholine and accelerated colon transit, resulting in a significant and substantial improvement in opioid-induced constipation in rats.^[50]^ The findings of the animal study suggested a necessity to optimize or search for effective parameters for SNS to be used for treating constipation.

More mechanistic studies and clinical trials with improved SNS methods are needed to bring the SNS therapy to benefit patients with constipation.

### SNS for irritable bowel syndrome

IBS is the most common functional gastrointestinal disease, affecting up to 20% of general population.^[51,52]^ It is defined as recurrent symptoms of abdominal pain or discomfort associated with alterations in bowel traits or habits, in the absence of any other diseases to account for the symptoms.^[53]^ It is classified as constipation-dominant IBS (IBS-C), diarrhea-dominant IBS (IBS-D) and IBS with a mixed bowel pattern (IBS-M). The prevalence of IBS in females is about 1.5 to 2 times of that in males.

The main pathophysiology of IBS includes visceral hypersensitivity (or altered pain perception), impaired brain-gut interaction, altered microbiota and altered gastrointestinal motility.^[53]^ Among these, visceral hypersensitivity is believed to be the major cause of abdominal pain or discomfort; visceral hypersensitivity is believed to be attributed to increased intestinal permeability and gut mucosal immune activation. Various medications have been used for treating symptoms of IBS based on its major pathophysiological mechanisms. Antidepressants (or called neuromodulators) and opioids are commonly used for treating abdominal pain in IBS.^[53]^

There were a few clinical studies on PubMed and Web of

Science exploring the effectiveness of SNS for visceral pain in patients with IBS. The earliest clinical study on the application of SNS for IBS was a temporary SNS study performed in 6 patients with IBS-D *via* an implanted SNS lead and an external stimulator.^[54]^ The 2-week open-label treatment resulted in a significant decrease in the score of IBS total symptoms (42%), quality of life (40% decrease) and abdominal pain (44%). The methodology (stimulation parameters and location) used was the same as that for treating fecal incontinence. In 2014, Fassov *et al*. published a randomized, controlled, crossover study of SNS in 26 patients with IBS-D or IBS-M.^[55]^ The patients underwent a temporary SNS study with a predefined positive effect and were then randomized to receive a two-month crossover treatment (one-month on and the other month off). A significant improvement was noted in the IBS symptom score and IBS-related quality of life with SNS-on in comparison with SNS-off in these highly selected patients with IBS. In 2019, the same groups published another SNS study in 21 patients with IBS-D or IBS-M.^[10]^ In this study, no temporary SNS was used to screen the patients. The SNS was performed in a randomized, double-blinded, placebo-controlled crossover fashion. The outcomes were compared between a two-week SNS-on period and a two-week SNS-off period. Improvement was reported in IBS symptoms (*P* = 0.057), pain (*P* = 0.02) and the number of daily bowel movement (*P* = 0.037).

Although these preliminary clinical studies showed a promising effect of SNS on IBS, more studies are needed to 1) optimize SNS methodology for treating IBS. In previous studies, the SNS method was simply copied from the SNS used for treating fecal incontinence. While this SNS method might be appropriate for treating certain symptoms of IBS, such as diarrhea due to its physiological similarity with fecal incontinence and overactive bladder, it may not be suitable or most suitable for treating abdominal pain that is the major symptom of IBS; 2) it was unknown by which mechanisms SNS was able to ameliorate symptoms of IBS; 3) more clinical studies are needed to prove the efficacy of SNS for treating IBS.

The potential analgesic effect of SNS on visceral pain was studied in a rodent model of visceral hypersensitivity. ^[56]^ In rats with instillation of acetic acid into the bladder, acute SNS was found to inhibit the arterial pressure response to colorectal distention. In pigs with increased permeability induced by 2,4,6-trinitrobenzenesulfonic acid (TNBS), acute SNS was reported to enhance mucosal repair by suppressing ZO-1 expression and its epithelial pericellular distribution, increased pFAK/FAK expression and inflammatory cytokines of interleukin (IL)-1β and IL-4.^[57]^ It should be noted that the SNS in these studies was performed using the parameters used for fecal incontinence and in animal models rather than patients with IBS.

In a recent rodent study, we performed a series of experimental sessions to optimize stimulation parameters of SNS in a rodent model of IBS.^[58]^ Colonic hypersensitivity was established in rats by intrarectal administration of acetic acid during the neonatal stage. The visceromotor reflex in response to graded colorectal distention was assessed by the abdominal electromyogram with sham-SNS and SNS of 6 different sets of stimulation parameters used in various applications of SNS. The most effective set of SNS parameters for suppressing the visceromotor reflex in response to colorectal distention was found to be as follows: 14 Hz, 330 μs and 40% motor threshold. SNS with this set of parameters substantially improved colonic visceral hypersensitivity mediated *via* the autonomic and opioid mechanisms. Clinical studies are needed to investigate whether this method of SNS is effective in treating abdominal pain in patients with IBS.

## SNS for Inflammatory Bowel Disease

### Inflammatory bowel disease

IBD, including Crohn’s disease and ulcerative colitis, is highly prevalent; severe IBD is difficult to treat.^[59]^ It was reported to affect 3 million people in the US in 2015^[60]^ and more than 0.3% of people in North America, Oceania and many European countries.^[61]^ A recent meta-analysis revealed that since 1990, incidence has been rising in newly industrialized countries in Africa, Asia, and South America.^[61]^ Persistent diarrhea, rectal bleeding, abdominal pain and weight loss are main symptoms of IBD. Although the exact pathogenesis of IBD is still unclear, IBD is believed to be attributed to the interaction of a number of factors, including genetic predisposition, environmental factors, intestinal barrier, and immune response.^[62]^

Pharmacological therapies are used to treat symptoms but cannot cure the disease. Current common medications for IBD include anti-inflammatory and immune-suppressive agents such as aminosalicylates, corticosteroids, immunoregulatory agents, antibiotics, anti–tumor necrosis factor alpha (anti-TNF-α), anti IL-12/IL-23, anti-integrin, Janus kinase (JAK) inhibitor, *etc*.^[63]^ Although these drugs have been reported to be effective in achieving induction and maintenance of remission, the clinical effectiveness is limited and serious side effects are not uncommon: about 30% of IBD patients may not respond to anti-TNF-α, and about 36% eventually lose response to the drug in the first year of treatment.^[64]^ Adverse events from the use of anti-TNF-α were reported to include infusion reactions, infection, and malignancies such as lymphomas and melanoma.^[65]^ Accordingly, more effective and safe therapies are needed.

### SNS for IBD: clinical studies

The SNS is not approved for treating ulcerative colitis or Crohn’s disease. However, it was performed in patients with fecal incontinence suffering from IBD-related symptoms. In one study, temporary (3 weeks) and permanent SNS were performed in 5 patients with fecal incontinence and Crohn’s disease-related anoperineal lesions. Continence was improved in all patients with both temporary and chronic SNS (up to 3 years). However, the patients did not have active Crohn’s disease at the time of inclusion and no changes in Crohn’s disease symptom score were noted during the study period.^[66]^ In another study, SNS was performed in a patient with fecal incontinence and refractory ulcerative proctitis. A 3-week SNS decreased both fecal incontinence score and ulcerative colitis disease activity score, and the improvement was sustained after 18 months of permanent SNS. Interestingly, improvement in rectal barrier permeability was also noted.^[67]^

Although the SNS was indicated for treating fecal incontinence in these cases series, the findings suggested a potential that SNS might also be used for treating intestinal inflammation.

### SNS for intestinal inflammation: animal studies

Inspired by the cholinergic ant-inflammatory pathway and the anti-inflammatory effects of VNS,^[68,69]^ a number of preclinical studies were recently performed to explore methods, effects and mechanisms of SNS for intestinal inflammation.

**Figure 3:**
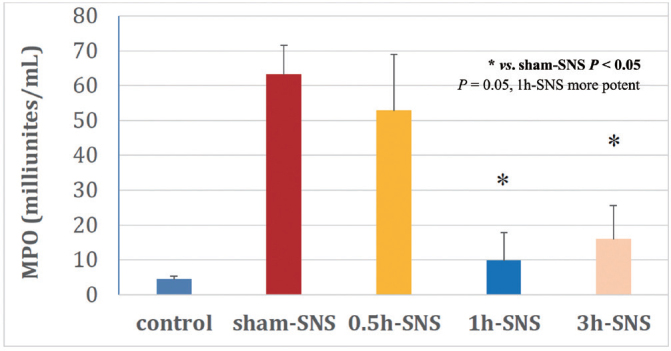
Effects of SNS with optimized parameters and different daily stimulation durations on colonic myeloperoxidase (MPO) activity in normal (control) and TNBS (2,4,6-trinitrobenzene sulfonic acid)-treated rats. Sham-SNS: no stimulation; 0.5 h-SNS: daily 0.5 h SNS. SNS: sacral nerve stimulation.

#### Methods of SNS for intestinal inflammation

In order to derive an effective method of SNS for intestinal inflammation, Zhang *et al*. performed a systematic study in a rodent model of IBD using different stimulation parameters, locations and daily treatment durations.^[11]^ First, two sets of stimulation parameters were compared in their inhibitory effects on colonic inflammation, one with 14 Hz, 210 μs and continuous stimulation used in SNS for fecal incontinence and the other with 5 Hz, 500 μs and intermittent stimulation (10s-on and 90s-off) that was shown to improve colonic inflammation with electroacupuncture at acupoint ST36.^[70]^ The intermittent stimulation of SNS with 5 Hz and 500 μs was found to be significantly more effective in reducing disease activity index score and improving inflammatory cytokines. Secondly, experiments were performed to optimize the daily SNS treatment duration. Among three treatment durations tested, 0.5 h, 1 h and 3 h, 1 h daily SNS was noted to be most appropriate as its effect on colonic inflammation assessed by myeloperoxidase (MPO) was significantly more potent than 0.5 h daily SNS but comparable with 3 h daily SNS (see Figure 3).^[11]^ Finally, the best stimulation configuration was derived: bipolar SNS was better than unipolar SNS and unilateral was superior to bilateral.

Consequently, the best SNS method for treating TBNS-induced colonic inflammation in rats was described as follows: Unilateral bipolar stimulation at S3 with a frequency of 5 Hz, pulse width of 500 μs, stimulation on-time of 10 s and off-time of 90 s, and current amplitude of 90% of motor threshold (typically < 1 mA).^[11]^

#### Anti-inflammatory effects of SNS in rats

**Figure 4:**
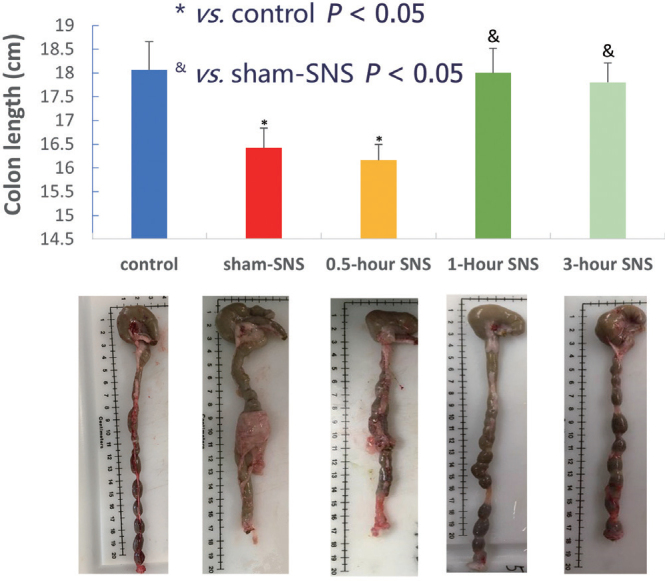
Effects of SNS on colon length rats. Control: regular rats without any treatment; sham-SNS: rats treated with TNBS and implanted with SNS electrodes without stimulation; 0.5/1/3-hour SNS: rats treated with TNBS and 0.5/1/3 hour daily SNS for a week; appearance of the colon at the end of the 7-day treatment. Modified from Zhang *et al*.^[11]^ SNS: sacral nerve stimulation; TNBS: 2,4,6-trinitrobenzene sulfonic acid.

The above-described method of SNS was shown to have inhibitory effects on colonic inflammation in two rodent models of intestinal inflammation induced by TBNS (one-time intrarectal administration) and dextran sodium sulphate (DSS, mixed in drinking water).^[12,14]^ As shown in Figure 4,^[11]^ the colon length was significantly reduced in TNBS treated rats (“sham-SNS”) in comparison with the normal rats (“control”) and the inflammation in the colon was clearly visible. The SNS increased the colon length and improved inflammation when it was delivered 1 h (“1-hour SNS”) or 3 h (“3-hour SNS”) daily for a period of 7 days but not 0.5 h (“0.5-hour SNS”) daily.

**Figure 5:**
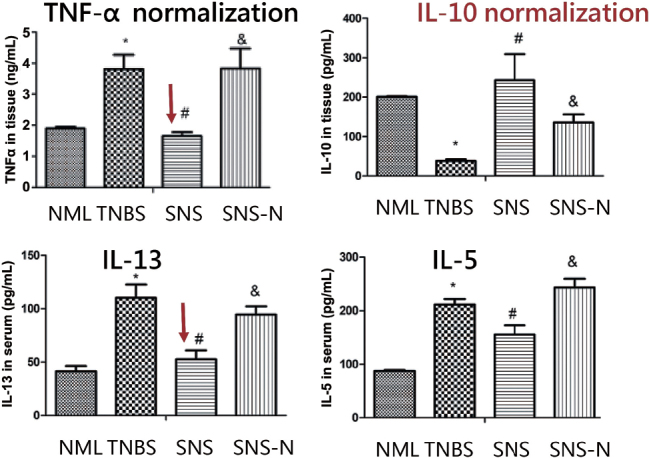
Effects of SNS on multiple inflammatory cytokines. top-left: Tumor necrosis factor-α (TNF-α) in colon tissues in various groups of rats: NML: normal rats without treatment, TNBS: rats treated with TNBS but not SNS; SNS: rats treated with TNBS and then SNS of optimized parameters; SNS-N: rats treated with TNBS and then SNS of parameters used for fecal incontinence; top-right: interleukin-10 (IL-10) in colon tissues; bottom-left: IL-13 in colon tissues; bottom-right: IL-5 in colon tissues. *P < 0.05, *vs*. NML; ^#^*P* < 0.05, *vs*. TNBS; ^&^*P* < 0.05 *vs*. SNS. Modified from Zhang *et al*.^[11]^ SNS: sacral nerve stimulation; TNBS: 2,4,6-trinitrobenzene sulfonic acid.

No different was noted in the colon length between 1 h and 3 h daily SNS.^[11]^ The effects of SNS on multiple inflammatory cytokines are shown in Figure 5.^[11]^ One of major pro-inflammatory cytokines, TNF-α was doubled in rats treated with TNBS (“TNBS”, top-left in the panel) in comparison with the normal control rats (“NML”). The treatment of SNS with the effective parameters (“SNS”) normalized the tissue TNF-α level, whereas the treatment of SNS with the parameters used for treating fecal incontinence (14 Hz, 210 μs and continuous stimulation) did not result in such an effect (“SNS-N”). Moreover, two other pro-inflammatory cytokines, IL-13 and IL-5 were also suppressed by the treatment of SNS. Most interestingly, the anti-inflammatory cytokine, IL-10 was substantially suppressed in rats treated with TNBS but normalized with SNS (top-right in the panel). These findings suggested multiple inflammatory cytokine mechanisms involved in the anti-inflammatory effect of SNS.

#### Neural pathways of SNS for intestinal inflammation

**Figure 6:**
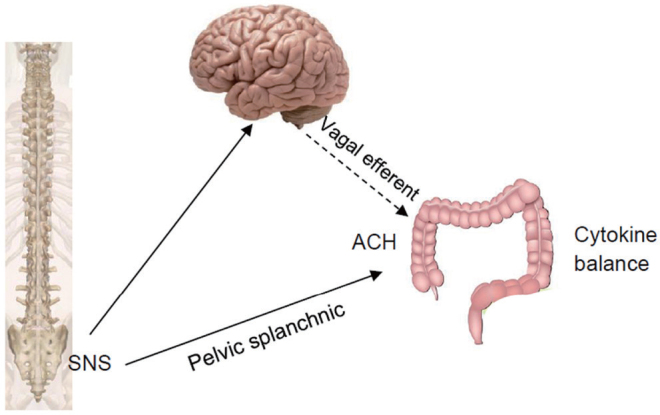
Neural pathways involved in the anti-inflammatory effect of SNS. SNS activates neurons in the nucleus tractus solitarius, resulting in an enhanced vagal efferent activity; acetylcholine (ACH) is released in the colon due to activation of vagal efferent nerve; ACH acts on a special receptor in macrophage to balance inflammatory cytokine release. SNS also acts on the colon *via* pelvic splanchnic nerve. SNS: sacral nerve stimulation.

Two neural pathways have been proposed and investigated on the anti-inflammatory effect of the SNS method. As shown in Figure 6^[13]^, one neural pathway is established based on the anatomical connection of the sacral nerve (to the colon. SNS is believed to activate sacral efferent, resulting in a release of acetylcholine in the colon and balancing the releases of inflammatory cytokines *via* the cholinergic anti-inflammatory pathway.^[68,71,72]^ The other neural pathway, the spinal afferent-brainstem-vagal efferent pathway, was proposed based on experimental findings.^[13]^ SNS was shown to activate neurons in the nucleus tractus solitarius assessed by c-Fos expression and resulted in an enhanced vagal efferent activity determined by the spectral analysis of heart rate variability. The SNS-induced release of acetylcholine in colon tissues was documented in TBNS-treated rats with SNS in comparison TBNS-treated rats with sham-SNS.^[13]^ Improvement in multiple inflammatory cytokines is shown in Figure 5.

The discovery of the spinal afferent - vagal efferent pathway mediating the SNS effects is of great clinical significance as this paves the road for SNS to alter functions of peripheral organs, such as the stomach and small intestine that are innervated with the vagal nerve but not the sacral nerve. Examples of such applications are presented in the following section.

#### Mechanisms of SNS for intestinal inflammation

In addition to the mechanisms involving inflammatory cytokines *via* the cholinergic anti-inflammatory pathway, SNS has also been shown to improve the intestinal mucosal barrier function.^[57,73,74]^ Using the same SNS method as described above, Tu *et al*. showed a reduction in colonic permeability in TNBS-treated rats assessed by the FITC-dextran test.^[75]^ In addition, the protein expressions of Zonula Occludens-1, Occludin, Claudin-1, and Junctional adhesion molecule-A in the colon tissue were significantly increased with the SNS in comparison with sham-SNS.

Mechanisms of SNS involved in the enteric nervous system were explored in a sperate study using the same SNS method and the same rodent model of intestinal inflammation;^[76]^ the percentage of choline acetyltransferase neurons in the colon tissue was decreased by TNBS but reversed by SNS, whereas the percentage of nitric oxide synthase (NOS) neurons in the colon tissue was increased by TNBS but decreased by SNS. In addition, SNS reduced the percentage of Th1 cells in the colon tissue that was increased by TNBS and increased the percentage of Treg cells in the colon tissue that was reduced by TNBS.

## Potential of SNS for Upper Gastrointestinal Motility Disorders

Gastrointestinal motility is critically important in the transportation, digestion and absorption of ingested foods and nutrients. Upper gastrointestinal motility disorders are common in functional dyspepsia, gastroparesis and chronic intestinal pseudo-obstruction.^[12,77]^ The parasympathetic nerve system, including the vagal and sacral nerves, plays an important role in the regulation of gastrointestinal motility. Anatomically, VNS is believed to enhance motility of the upper gastrointestinal organs; whereas, SNS is believed to only after motility of the lower gastrointestinal organs. Therefore, SNS had never been explored for its potential in treating motility disorders of the upper gastrointestinal organs. According to the newly observed spinal afferent and vagal efferent pathway of SNS, SNS is believed to be capable of altering upper gastrointestinal functions although there is no direct anatomical innervation of the sacral nerve to the upper gastrointestinal organs as shown in the following two recent studies.^[15,16]^

### Effects of SNS on gastric relaxation

Food ingestion-induced relaxation of the proximal stomach is critically important and an integrative part of gastric functions; it allows the stomach to accommodate ingested food without causing an increase in the stomach pressure that might generate a feeling of bloating. This physiological function is referred to as gastric accommodation and accomplished by the activation of the vagovagal reflex and release of nitric oxide and vasoactive intestinal peptide in the proximal stomach. Gastric accommodation is impaired in patients with functional dyspepsia and gastroparesis that affect millions of people in the US.^[78,79]^ Impaired (reduced) gastric accommodation is associated with increased sensation of bloating and early satiety as well as weight loss.^[80]^ Medical treatment for impaired gastric accommodation in patients with functional dyspepsia and gastroparesis is difficult: although muscle relaxants could be used to relax the proximal stomach to increase gastric accommodation, these medications would impair antral contractions and delay stomach emptying, and therefore could worsen symptoms of dyspepsia and gastroparesis. Accordingly, it is of great clinical significance to develop a novel therapy that relaxes the proximal stomach but does not inhibit antral contractions or delay stomach emptying.

A recent rodent study has shown an enhancive effect of SNS on gastric accommodation.^[15]^ Using the same stimulation parameters of SNS for intestinal inflammation, acute SNS was shown to reduce fundic tone and increase gastric accommodation in regular rats with a concurrent increase in vagal efferent activity, suggesting a vagal efferent mechanism. Moreover, the effects were blocked by N-nitro-L-arginine methyl ester (L-NAME), a nitric oxide synthase inhibitor, demonstrating a nitric oxide mechanism. To prove the spinal afferent pathway involved in the SNS effects, the authors also showed an increased expression of c-Fos in the nucleus tractus of solitarius with SNS in comparison with sham-SNS.

### Ameliorating effects of SNS on gastrointestinal dysrhythmia

**Figure 7:**
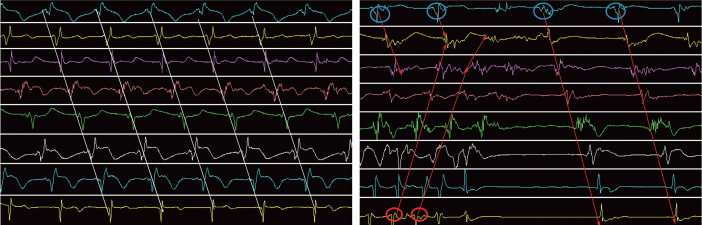
Gastric slow waves recorded from a dog via chronically implanted electrodes on the gastric serosa. Top channel: corpus; bottom channel: distal antrum. Left: normal slow waves with a regular frequency (about 5 waves/min), propagating from the corpus to the distal antrum. Right: Abnormal slow waves with a normal pacemaker in the corpus (top channel) and an ectopic pacemaker firing at a higher frequency in the distal antrum. Reproduced from^[83]^.

Once the food is ingested into and stored in the proximal stomach, it is mixed, grinded and emptied from the stomach to the duodenum by antral peristalsis. The antral peristalsis is the distally propagated antral contraction. Antral contraction is regulated by gastric pace-making activity similar to cardiac contraction and cardiac pacemaker. The basic rhythm of gastric pace-making activity is called the gastric slow wave due to its slow rhythmicity (3 cycles/min in humans and about 5 cycles/min in rats). The gastric slow wave determines the frequency and propagation of antral contractions.^[81,82]^ Under normal conditions, the gastric slow wave propagates distally from the corpus to the pylorus and the stomach is in a coordinated manner (see Figure 7, left panel).^[83]^ Under abnormal conditions, however, there could be ectopic pacemakers in different areas of the stomach with or without the normal pacemaker. Figure 7 (right panel) shows an ectopic pacemaker in the distal antrum firing at a frequency higher than the normal rhythm and propagating aborally from the antrum to the corpus; meanwhile there were also normal gastric slow waves originated from the corpus and propagate distally. In this case, the stomach was not in a coordinated fashion. Previous studies have shown that abnormal gastric slow waves were associated with delayed gastric emptying.^[84]^

**Figure 8:**
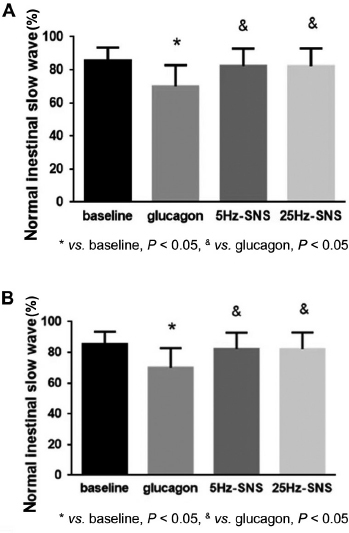
Effects of SNS on gastric and small intestinal slow waves impaired by the treatment of glucagon. A. Percentages of normal gastric slow waves for baseline, glucagon, glucagon + 5 Hz SNS, and glucagon + 25 Hz SNS. B. Percentages of normal intestinal slow waves for baseline, glucagon, glucagon + 5 Hz SNS, and glucagon + 25 Hz SNS. *P < 0.05 *vs*. baseline. ^&^*P* < 0.05 *vs*. glucagon. Reproduced from Wang *et al*..^[16]^ SNS: sacral nerve stimulation.

In supporting the spinal afferent and vagal efferent pathway of SNS, another recent rodent study showed SNS-induced improvement of gastric slow waves impaired by rectal distention and glucagon.^[16]^ As shown in Figure 8A,^[16]^ the percentage of normal gastric slow waves was substantially reduced after glucagon injection but increased to the normal level with acute SNS of the same parameters used for treating intestinal inflammation. Similar ameliorating SNS effects were also observed in small intestinal slow waves (Figure 8B): the glucagon-induced decrease in the percentage of normal intestinal slow waves was normalized by SNS of the same parameters.

### Potential of SNS for upper gastrointestinal disorders

The findings of the above preclinical studies might suggest potential application of SNS for functional dyspepsia and gastroparesis. Both functional dyspepsia and gastroparesis have been reported to have impaired gastric accommodation and antral hypomotility. In the above two rodent studies, the SNS was shown to improve not only gastric accommodation but also antral slow waves, a surrogate of antral motility. These unique effects make SNS a possibly viable therapy for treating functional dyspepsia and gastroparesis, warranting further clinical studies.

## Discussion and Perspectives

While SNS has potential for treating various gastrointestinal diseases, the treatment of fecal incontinence is the only clinical application that has received regulatory clearance. Although its mechanisms of action remain to be elucidated, the clinical efficacy of SNS for fecal incontinence is established. The perspective on the application of SNS is to further improve its efficacy by exploring mechanisms of SNS involved in the ameliorating effect of SNS on fecal incontinence and its efficiency by optimizing the treatment regimens, such as daily stimulation duration, and stimulation parameters, *etc*.

Constipation is of high prevalence and affects quality of life substantially. SNS for constipation has been explored in numerous clinical studies. However, its clinical efficacy has not been established. Several systematic reviews and controlled studied have demonstrated ineffectiveness of SNS for constipation using the conventional method: a stimulation frequency of about 10-20 Hz, pulse width of 200-300 μs and duty-cycle of 100% (continuous stimulation). Further methodological development and refinement are needed to make SNS effective for constipation, such as optimization of stimulation parameters, including frequency, pulse width and duty cycles as well as appropriate intermittent stimulation. In order to do so, it is critically important to explore mechanisms of SNS on colon transit and rectal sensation as well as other pathophysiological factors of constipation.

IBS is the most prevalent functional gastrointestinal disease, affecting millions of people in the US. Since the major symptom of IBS is abdominal pain and neuromodulation is known to improve pain resulting from various etiologies, there is a great potential for SNS to treat abdominal pain in patients with IBS although current clinical evidence is limited. Basic preclinical and mechanistic studies are needed to derive effective stimulation parameters and regimens for the treatment of visceral hypersensitivity in animal models of IBS. Due to differences in pathophysiology of IBS among different subgroups, different SNS methods may be needed for treating constipation-dominant IBS and diarrhea-dominant IBS. Although IBS is a prevalent disease, a large proportion of patients suffer from only mild to moderate symptoms. Accordingly, it also remains to be determined whether the invasive SNS therapy will be well received by patients and healthcare professionals, which we believe would be determined by the ratio of benefits (efficacy) and risks/costs.

Although it is too early to predict, IBD might present a unique opportunity for SNS because of the following: (1) IBD is highly prevalent and affects millions of people around the world;^[60]^ (2) a large proportion of patients with IBD have moderate to severe symptoms that cannot be ignored; moreover, patients with IBD have an increased risk of cancer development;^[85]^ (3) medical therapies are expensive and the yearly medical cost for treating moderate/severe IBD using infusion therapy (infliximab or vedolizumab) is about 40,000 USD per year^[86]^ that is substantially more expensive than the neuromodulation therapy. However, currently, only preclinical studies and a few case reports are available to suggest the therapeutic potential of SNS. Clinical studies are warranted to explore the efficacy of the proposed SNS for treating patients with IBD.

In summary, SNS for fecal incontinence is a well-established clinical therapy. However, the current method of SNS is ineffective for treating constipation. Further methodological development and randomized clinical trials are needed to explore potential applications of SNS for IBS and IBD.
